# Fruit classification using attention-based MobileNetV2 for industrial applications

**DOI:** 10.1371/journal.pone.0264586

**Published:** 2022-02-25

**Authors:** Tej Bahadur Shahi, Chiranjibi Sitaula, Arjun Neupane, William Guo

**Affiliations:** 1 School of Engineering and Technology, Central Queensland University, North Rockhampton, QLD, Australia; 2 Department of Electrical and Computer Systems Engineering, Monash University, Melbourne, VIC, Australia; Universita degli Studi di Perugia, ITALY

## Abstract

Recent deep learning methods for fruits classification resulted in promising performance. However, these methods are with heavy-weight architectures in nature, and hence require a higher storage and expensive training operations due to feeding a large number of training parameters. There is a necessity to explore lightweight deep learning models without compromising the classification accuracy. In this paper, we propose a lightweight deep learning model using the pre-trained MobileNetV2 model and attention module. First, the convolution features are extracted to capture the high-level object-based information. Second, an attention module is used to capture the interesting semantic information. The convolution and attention modules are then combined together to fuse both the high-level object-based information and the interesting semantic information, which is followed by the fully connected layers and the softmax layer. Evaluation of our proposed method, which leverages transfer learning approach, on three public fruit-related benchmark datasets shows that our proposed method outperforms the four latest deep learning methods with a smaller number of trainable parameters and a superior classification accuracy. Our model has a great potential to be adopted by industries closely related to the fruit growing and retailing or processing chain for automatic fruit identification and classifications in the future.

## Introduction

Automatic fruit classification is an interesting problem in the fruit growing and retailing industrious chain because it can help the fruit growers and supermarkets identify different fruits and their status from the stock or containers so as to improve production efficiency and hence business profit. Therefore, intelligent systems using computer vision and machine learning methods have been explored for fruit defect identification, ripeness grading, and categorization in the past decade [1–3].

In automated fruit classification, two major techniques, traditional computer-vision based methods and deep learning-based methods, have been researched. The traditional computer vision-based methods first extract the low-level features and then perform image classification using the traditional machine learning method, whereas the deep learning-based methods extract the features effectively and perform an end-to-end image classification [4]. In the traditional computer vision and image processing methods, imagery features, such as texture, color, and shape, have been used as inputs for fruit classification. For example, Muhammad et al. [5] proposed an automatic classification of ‘Date’ fruit using hand-crafted features. A classification accuracy of 98% was reported with a support vector machine (SVM) classifier. A cucumber shape classification system based on shape features, such as area, perimeter, eccentricity, extent, roundness, compresses, width-non-homogeneity and centroid-non-homogeneity, was proposed by Kheiralipour et al. [6] in which the neural network (NN) was trained to classify cucumber into two shapes: desirable and undesirable, which produced a classification accuracy of 97.10%.

With the rise of deep learning (DL)-based methods in various disciplines, such as stock market [7], scene images [8], biomedical [9], and privacy [10], researchers have been working towards the development of deep learning-based methods for the fruits classification problem, using the transfer learning (TL) approach mostly [11, 12]. For example, Bhole et al. [11] explored the applicability of the TL approach in fruit classification utilizing a pre-trained model, called SqueezeNet [13], to classify Mangoes into three grades: extra class, class I and class-II. They evaluated the proposed model for the classification of Mango ripeness and size, which achieved an accuracy of 93.33% and 92.27% on the RGB image dataset and thermal dataset, respectively. In a similar study, Xiang et al. [14] achieved a classification accuracy of 85.12% using the TL approach on lightweight MobileNetV2 [15] model with a dataset of 3,670 images for five fruits: apple, banana, carambola, guava and kiwi.

In addition to the transfer learning, the deep neural networks from scratch were also proposed for fruit classification in literature [4, 16, 17]. For example, a large fruit dataset was introduced by Mureşan et al. [16] for fruit classification using DL models. This dataset, also known as Fruit-360 dataset, which consists of 28,736 training images and 9,673 testing images. A convolutional neural network (CNN) with four convolutional layers, each followed by a max-pooling layer, fully connected layer and finally, softmax layer, was used for the fruit classification. They achieved an accuracy of 95.23% using some data augmentation strategies such as flip, hue/saturation changes and gray-scale.

Among the aforementioned techniques, computer vision-based methods are used to classify the diversity of same fruit species as in [5] and [6], which may not be robust for different kind of fruit classification, whereas the deep learning-based methods are used to classify variety of fruits [3, 4, 17]. However, existing DL-based methods still have two main limitations. Firstly, such methods mostly require a large number of trainable parameters although they claim that their models to be lightweight architectures. As a result, this may be infeasible to deploy on lightweight environments such as mobile/edge computing platforms. Secondly, the performances of these models are dependent on the number of classes and the number of available datasets.

To overcome the existing limitations, we propose a novel lightweight deep learning model based on the MobileNetV2 [15], which is known to have a lightweight architecture compared to other pre-trained models such as VGG-16 [32]. Although our proposed model utilizes two well-established concepts (convolution and attention), we believe that, to the best of our knowledge, combination of these two concepts for lightweight architecture is the first work in fruit classifications. The combination of the convolution and attention modules is expected to work complementarily to each other to achieve better performance in fruit classification. The convolution module captures the convoluted image features, whereas the attention module captures the salient regions in the image. To evaluate the efficacy of our proposed method, we have conducted experiments on three different publicly available datasets, which show that our method imparts the stable performance.

The main contributions of the paper are as follows:.

(i) We propose a novel deep learning method based on the existing MobileNetV2 model along with the attention approach. The attention module captures the salient region in fruit image whereas the convolution module captures the activated regions achieved by Rectified Linear Unit (ReLU) function over the fixed kernel size. The combination of convolution and attention modules helps discriminate the diverse classes of fruits as they are complementary to each other.
(ii) Our proposed method requires a smaller number of trainable parameters as we leverage the pre-trained weights for all layers of MobileNetv2 architecture. This makes our model more suitable to deploy on resource-constrained devices.
(iii) Our proposed model can be trained and deployed in an end-to-end fashion, which avoids the separate feature extraction and classification steps as in traditional machine learning approach.
(iv) We validate our model utilizing three different fruit datasets to confirm the robustness of our model. Experimental results show that our method has stable performances on all fruit datasets and also outperforms other latest DL-based methods.

Our paper is organized as follows. Section “Related work” summarizes the existing works related to fruit image classification. Furthermore, Section “Proposed method” explains our method and its components. Similarly, Section “Experimental setup” discusses the datasets and implementation details. Furthermore, Section “Result and discussion” compares our method with existing works using widely-used evaluation metrics. Section “Conclusion” concludes the paper with future works.

## Related work

In recent years, researcher in agriculture and food industry are interested in applying machine learning and deep learning methods for various applications such as fruit detection [18], fruit classification [19], yield estimation [20], fruit grading [21], disease classification [22], and so on. In this section, we summarize some of the recent methods that have been used widely in fruit related applications such as classification and detection. We discuss these methods under two subsections: low level feature-based methods and deep learning methods.

### Low level feature-based methods

A fruit classification system based on image features such as color, shape and texture was proposed by [19]. These fruit images’ features dimensions were first reduced using principal component analysis (PCA) [23] and then fed into the classification algorithms such as fed-forward neural network (FNN) and support vector machine (SVM). They experimented with 1,653 color fruit images from the 18 different fruit classes and reported the highest accuracy of 88.2% with SVM. However, the fruit images used in the experiments are fine-grained and they did not include the fruits in various conditions such as sliced, dried, and partially covered. Authors in [24] used a Random Forest (RF) method for three kinds of fruits classification: Strawberry, Apple, and Oranges. They used traditional feature extraction approaches to extract various features such as color, shape and scale invariant features (SIFT). Their results show that fruits with different shapes are difficult to classify than the fruits with the similar shapes. However, their experiments used the limited number of fruit samples (137 fruit images in total). The combination of machine learning algorithms and color space for Cape gooseberry ripeness classification was attempted in [25]. They used 925 fruit samples for model training and validation and reported that SVM produced the best F1-measure (70.14%) among twelve different machine learning classifiers. However, their method suffers from a limited performance and small dataset size. Their method is limited to the PCA-based feature fusion and have not considered the other feature fusion methods such as fusion of score or decisions. A local binary pattern based features were used in bark texture classification using multilayer neural network [26]. Similarly, a tomato classifier system was proposed in [21] using traditional image features such as color shape and size. A total of 100 tomato images were considered for classifying into large, small, and medium classes along with four grades. They achieved a mean grading accuracy of 90.7%. A similar study on post-harvest banana classification using machine learning method such as Artificial Neural Network (ANN), SVM, and RF was investigated in [27]. They considered four classes of banana: “extra class”, “class I”,“class II” and “reject” and collected a banana dataset of 1,164 instances. The reported results show that RF classifier with an accuracy of 94.20% outperforms the remaining classifiers.

Furthermore, there are a few studies that use images other than RGB such as Near infrared (NIR), and multispectral images. Authors in [28] proposed a in-field leaf spectroscopy-based method for Grapevine varieties classification. For this, they collected the grapevines’ leaves and took the NIR spectra measurement of individual leaf from 20 types of vines. These spectra were then fed into two machine learning algorithms: support vector machine (SVM) and Artificial Neural Network (ANN). The overall classification accuracy produced by their method was 87.25%. Also, a strawberry ripeness classification using multispectral imaging of 17 bands was proposed in [29], where the PCA was applied to reduce the dimension of multispectral images’ features. Then, these feature were used in three classifiers: Partial least square (PLS), SVM and ANN. Their results show that it achieved a higher classification accuracy of 100% when using the SVM classifier. The VIS (visual wave-bands) part of spectra were the main contributors in ripeness classification in their method.

### Deep learning-based methods

The deep learning (DL) methods are evolved along with the advancement of artificial neural network (ANN), which were inspired from human brain. Basically, DL-based method consists of a larger neural network having a numbers of layers, nodes, and activation functions [30]. Recently, deep learning methods become very popular and widely used in image classification tasks. Many works [3, 4, 17, 31] reported the promising results on fruit image classification task using deep learning methods. For example, Rojas et al. [17] proposed a lightweight CNN model for the classification on Fruit-360 dataset, which showed that the performance of CNN is increased by including additional features such as Red-Green-Blue (RGB) color and histogram. The highest accuracy was 93% for fruit classification in their method. However, the dataset used in their work consists of a limited number of fruits classes for Banana, Orange, and Apple. Furthermore, Joseph et al. [4] proposed a CNN model for fruit classification on the Fruit-360 dataset, which achieved an classification accuracy of 94.35%. And Hossain et al. [3] proposed a lightweight CNN model and compared it with fine-tuned VGG-16 model [32] for fruit classification on two datasets. Their method achieved a classification accuracy of 99.49% and 85.43% for their dataset-1 and dataset-2, respectively. In the meantime, Femling et al. [31] developed the system to classify fruits in retail store by capturing video with installed camera. They used two convolutional neural networks: InceptionV3 [33], and MobileNet [34] to detect and classify fruits and vegetable on video. The highest accuracy of 76% was achieved with InceptionV3 for ten-class fruit dataset. Furthermore, Chakraborty et al. [35] employed the MobileNetV2 model [15] with max-pooling and average-pooling to identify the rotten fruits. The highest accuracy of 94.97% was reported with Max-pooling operation. However, their method didn’t evaluate other standard CNNs with a higher number of classes. Recently, a oil Palm fruit ripeness classification was conducted by Herman et al. [36] with DenseNet model [37]. The evaluation of their method on a dataset of 400 images of oil palm fruits with 7 levels of ripeness produced a classification accuracy of 86%. Similarly, Saranya et al. [2] implemented a Banana ripeness classification system using lightwight MobileNetV2 and compared it with NASNetMobile [38] model. The MobileNetV2 [15] model outperformed NASNetMobile [38] with an accuracy of 96.10%. Furthermore, date fruit classification system for harvesting robot was developed in [39] using two deep learning models: AlexNet [40] and VGG-16 [32]. Here, the AlexNet is a lightweight architecture with smaller size and lower depth, whereas the VGG-16 is a deeper architecture. They used the transfer-learning and fine-tuning strategy with two pre-trained deep learning models (AlexNet and VGG-16) on ImageNet dataset for the fruit image classification. Their results show that the VGG-16 model has the highest accuracy of 99.01% during the fruit classification.

In addition, a lightweight model was proposed by Xiang et al. [14], which was based on transfer-learning approach using MobileNetV2. Their model considered its applicability in low-power and limited resource devices. They also added an extra convolution and a dropout layer on the top of base MobileNetV2 architecture. The MobileNetV2 pre-trained on ‘ImageNet’ dataset was used as a feature extractor, which was then fine-tuned with softmax layer on five-class fruit dataset with 3,213 training images. An accuracy of 85.12% was reported on fruit images (457 images) in validation set. However, this study is limited to five different types of fruits and has a limited number of samples. Similarly, Bahera et al. [41] conducted a non-destructive Papaya maturity classification using traditional machine learning method such as KNN, SVM, Naive Bayes, and transfer-learning with various pre-trained CNNs such as ResNet101, ResNet50, ResNet18, VGG-19, VGG-16, GoogleNet and AlexNet. They reported the highest accuracy for maturity classification (100%) when using pre-trained model, VGG-19 in comparison to other models. Nevertheless, due to the small training samples (only 300 fine papaya images), their method seem to be over-fitted and couldn’t be generalized for other unseen fruits samples. Besides fruit classification, a few studies attempted to classify the fruit diseases using computer vision and machine learning in the literature [22]. A citrus diseases classification was done using two lightweight pre-trained models: SqueezeNet [13] and MobileNetV2 [15], which was proposed by Khan et al. [22]. Their results show that the SqueezeNet model outperforms the Mobilenetv2 with an accuracy of 96% while classifying the features extracted from the corresponding pre-trained model for citrus disease using SVM classifier.

Recently, transformer-based deep learning methods, which are widely used for natural language processing (NLP), have been investigated for computer vision tasks such as image classification [42]. Since, the transformer are based on pixel-wise attention mechanism rather than convolution operation as in CNN [43], their use in computer vision task is still not mature enough. Also, a few works that employed transformers for image classification task reported that transformers outperform the CNN when they have enough training dataset [42]. Also, the availability of pre-trained models for various CNNs in comparison to vision transformer makes them more accessible for image classification task.

## Proposed method

**Fig 1:**
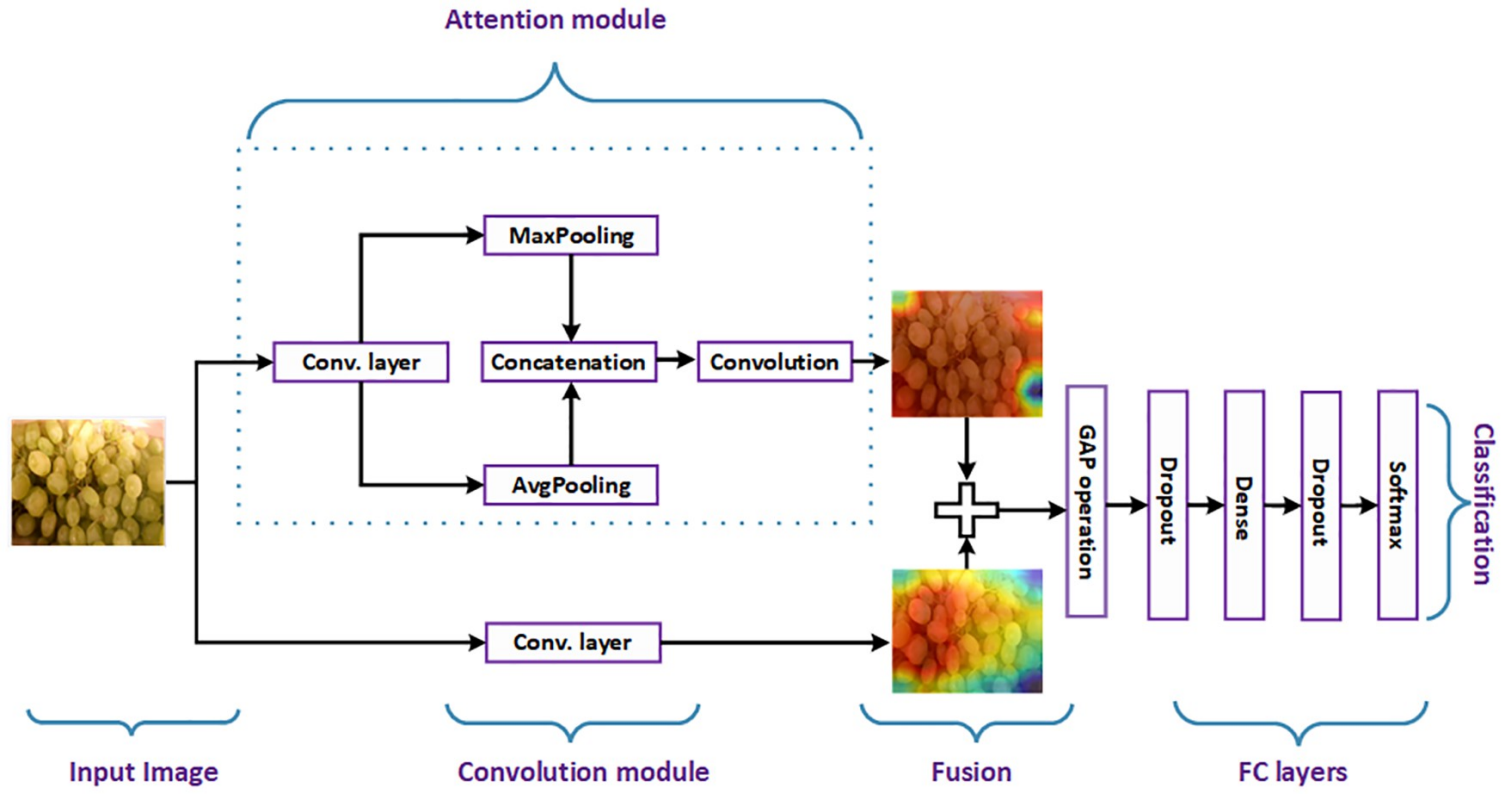
Overall block diagram of our proposed method for fruits image classification. Note that GAP stands for Global Average Pooling to convert the 3D features to 1D features and Conv. layer denote the last convolution layer of MobileNetV2 used in our work. Note that the fruit image used here is Republished from [44] under CC BY license, with permission from [Georg Waltner], original copyright[2022].

Our proposed method consists of six components: Preprocessing; the convolution module; the attention module; fusion of convolution and attention modules; the fully-connected layers; and classification. The overall flow of our method is presented in Fig 1.

### Preprocessing

Since our proposed method is based on MobileNetV2 [15] architecture, the input images must be with the specific size by rescaling. The use of the same input size as that used in pre-trained MobileNetV2 model helps produce highly discriminating features from images. Thus, all input images are resized to 224 by 224 within the pixel value [-1, 1]. We employ the online data augmentation parameters so as to compare our results with the existing outcomes (Table 2).

### The convolution module

Convolutional neural networks (CNN) have boosted the performance of image recognition tasks [8, 9, 45] and been a dominant network structure in deep learning technology. In other words, the use of CNN for image recognition and classification is not only becomes a trend in fruit classification [3] but also in other domains such as biomedical image analysis [9], scene images recognition [8], remote sensing image analysis [45] and so on. Thus, convolutional operations are major contributors in computer vision tasks but they are computationally expensive when the network structure goes deeper and larger as in VGG-16 [32], and InceptionV3 [33]. The MobileNetV1 [34] model brought the idea of depth-wise separable convolution, which divides the convolution into two sub-tasks: a depth-wise convolution that filters the input and a point-wise convolution (1 × 1) that combine these filtered values to create new features. The complete architecture of MobileNetV1 had a regular (3 × 3) convolution layer followed by 13 depth-wise separable convolution blocks [34]. The MobileNetV2 [15] model added the expand layer, residual connections and projection layers in addition to depth-wise convolution layers known as a bottleneck residual block. The expansion convolution layer (1 × 1) expands the number of channels according to the expansion factors whereas the projection layer reduces the number of channels into a tensor of lower channels. The residual connection helps the flow of gradients through the network. Here, each convolution layer is followed by the batch normalization and ReLu6 activation layer, ReLu6 being a variant of ReLu activation function limited to maximum size of 6. The complete MobileNetV2 architecture consists of 17 such bottleneck residual blocks followed by a regular (1 × × 1) convolution, a average pooling layer, and a classification layer [15] (refer to Table 1).

**Table 1:** The complete network structure of MobileNetV2.

Input Shape	Operator	t	c	n	s
224 × 224 × 3	conv2d	-	32	1	2
112 × 112 × 32	bottleneck	1	16	1	2
112 × 112 × 16	bottleneck	6	24	2	2
56 × 56 × 24	bottleneck	6	32	3	2
28 × 28 × 32	bottleneck	6	64	4	2
14 × 14 × 64	bottleneck	6	96	3	1
14 × 14 × 96	bottleneck	6	160	3	2
7 × 7 × 160	bottleneck	6	320	1	1
7 × 7 × 320	Conv2d 1 × 1	-	1280	1	1
7 × 7 × 1280	avgpool 7 × 7	-	-	1	-
1 × 1 × 1280	conv1d 1 × 1	-	k	-	

Note that t, c, n and s denotes the expansion factor, output channels, number of repetitions, and stride size respectively.

**Fig 2:**
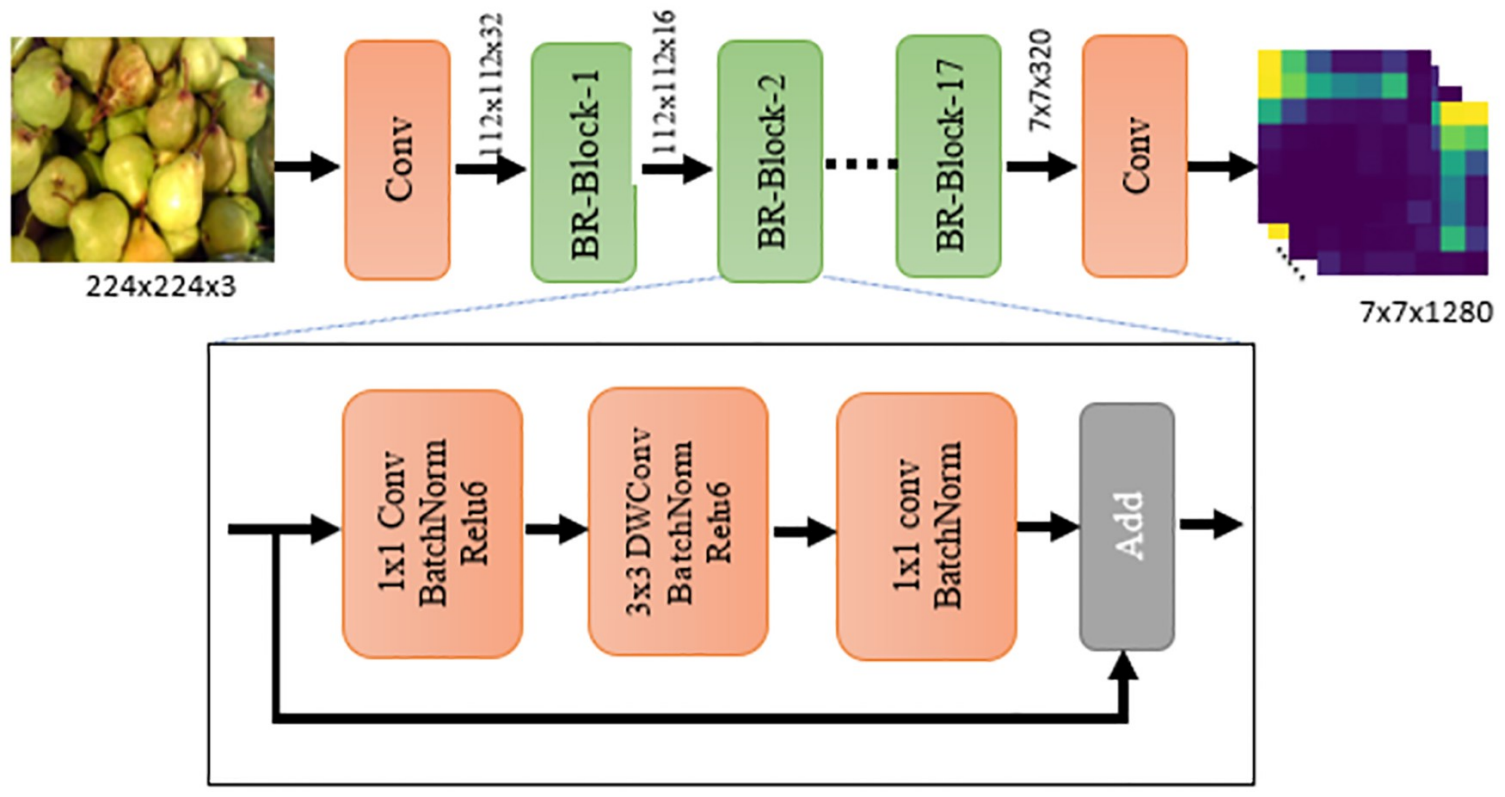
Pre-trained MobilenetV2-architecture used as convolutional module in this work [15]. Please note that *n*^*th*^ bottleneck residual block is denoted as ‘BR-Block-N’ in Fig 2. Note that the fruit image used here is Republished from [44] under CC BY license, with permission from [Georg Waltner], original copyright[2022].

We use the MobileNetV2 pre-trained with ‘ImageNet’ dataset as a backbone network. We extract the convolution feature map generated from the last residual block followed by the convolution layer of MobileNetV2 as shown in Fig 2. The lower-level residual blocks give the feature maps with a smaller size. These blocks do not capture the high-level clues for image recognition as a whole so they are not relevant in our work. During model design and training, we freeze all the layers up to the convolution layers that produce 3D feature map of size 7 × 7 (refer to Fig 2). This backbone network acts as convolutional feature extractor in our study. Mathematically, it is shown in Eq (1) as below:

$$\begin{array}{c} F1(I)=Conv(I), \end{array}$$ (1)

where F1(I) denotes the convolution feature for input image, *I*.

### The attention module

The attention network is inspired from the attention mechanism of human brain i.e., while observing images, people only focus their attention on important clues rather than looking at every detail on the object. As more researchers started working on attention mechanism, various models of attention evolved out [46]. Here, we are more interested in attention mechanism that not only points us where to focus on but also represents the spatial relationship of visual clues in the fruit images. Such attention module has been studies extensively in literature [46, 47]. Our attention module is based on convolutional block attention module (CBAM) proposed by Woo et al. [46]. In CBAM, it has two modules: channel attention and spatial attention modules. The channel attention module tries to capture “what” is important in the given images, whereas the spatial attention module focuses on ‘where’ or which part of an image is important (spatial). To focus on where is an informative part in the fruit image, the implementation of our attention module follow the spatial attention approach. Firstly, we perform the max-pooling and average pooling on the input from the convolution module. Secondly, the max-pooled tensor and average-pooled tensors are concatenated as suggested by Woo et al. [46]. Finally, convolution with a filter size of (7 × 7) is used to activate the visual clues in images with the sigmoid activation function. The overall steps for attention module are summarized in Eqs (2) and (3)

$$\begin{array}{c} F2(I)=[AvgPool(F);MaxPool(F)] \end{array}$$ (2)

Where *AvgPool*(*F*) ∈ *R*^*H*×*W*×1^ represents average pooling operation and *MaxPool*(*F*) ∈ *R*^*H*×*W*×1^ represents max-pooling operation. The concatenation of these two feature map on third dimension, produce a feature map, *F*2(*I*) ∈ *R*^(*H*×*W*×2)^.

$$\begin{array}{c} F3\left(I\right)=\sigma (f^{7\times 7}\left(F2\left(I\right)\right) \end{array}$$ (3)

where *σ* is a sigmoid activation function. *f*^7×7^ represent convolution operation of filter size (7 × 7), and F3(I) represents the attention features, which is *H* × *W* × *C* sized tensor with height (H), width (W), and depth (C).

### Fusion of convolution and attention modules

The feature maps acquired from convolution and attention modules are fused using a simple concatenation feature fusion approach as suggested by Sitaula et al. [48] to obtain a combined feature map. We choose a simple concatenation fusion approach rather than other methods such as the min, max, and sum because the two feature maps contains different properties of an image. Also, it is computationally cheaper than other methods such as the bilinear approach [49], which performs the product operation of tensors. The concatenation of these two features results in a single feature tensor of *H* × *W* × 1281 dimension. Mathematically, the concatenated resultant feature tensor *T*(*I*) for an image I is defined as in Eq (4).

$$\begin{array}{c} T(I)=[F1(I),F3(I)], \end{array}$$ (4)

where *F*1(*I*) ∈ *R*^*H*×*W*×1^ and *F*2(*I*) ∈ *R*^*H*×*W*×1280^ are 3D tensors with same height (H) and width (W), which allow them to concatenate on third dimension to produce 3D tensor *T*(*I*) ∈ *R*^*H*×*W*×1281^.

### The fully-connected (FC) layers

After the feature fusion, we use various fully connected (FC) layers to convert 3D tensor into one dimensional (1D) feature vector. These FC layers includes the global average pooling layer, dense layer, and dropout layer as depicted in Fig 1. We fix dense layer to 128 units with ReLu activation function and dropout rate to 0.5.

### Classification

**Fig 3:**
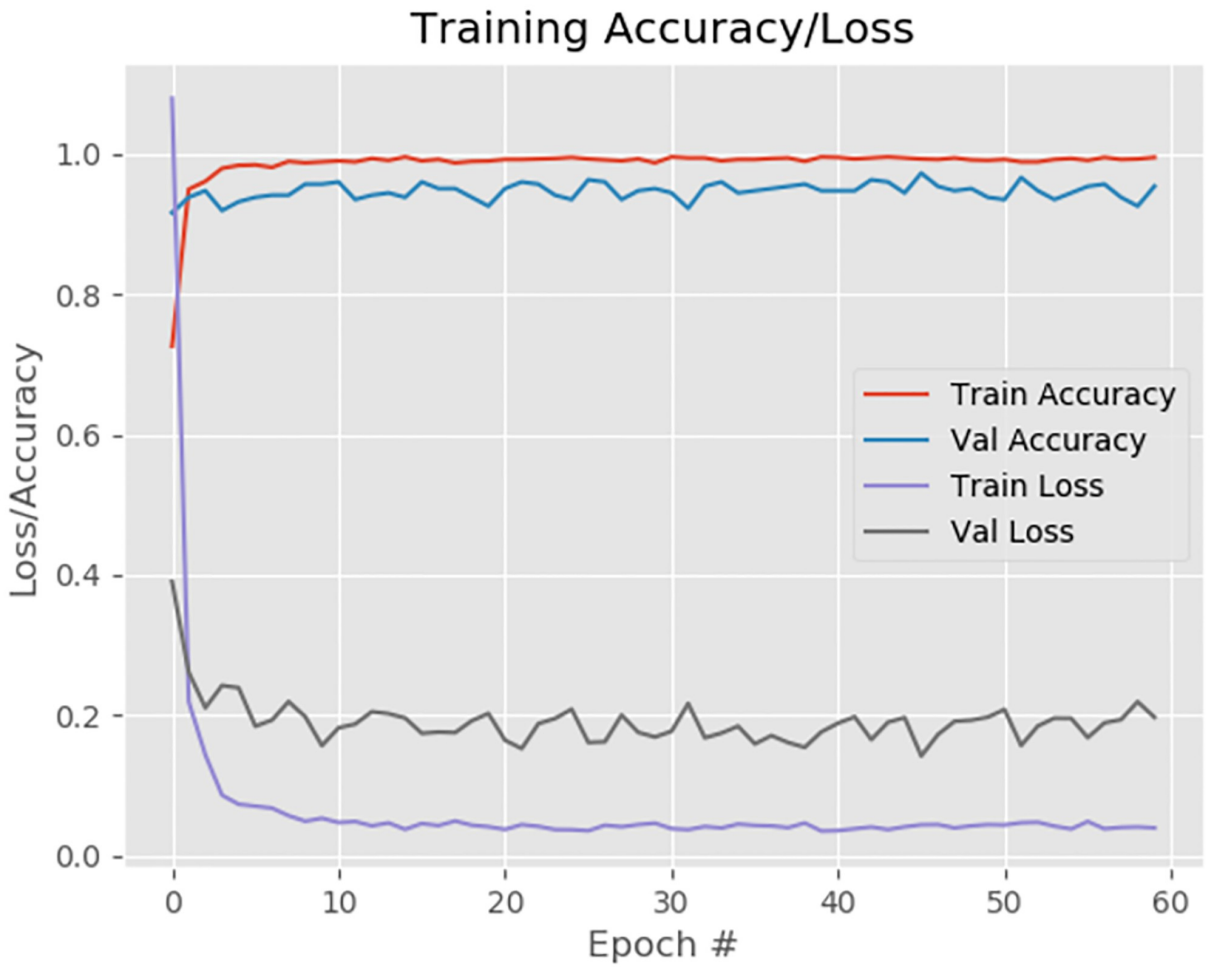
Model training accuracy and loss per epoch of our model on first set of D1.

The feature vector obtained from the final FC layer is fed into softmax layer to get the desired categories in the form of multinomial distribution. The softmax activation function normalizes the output of a previous dense layer into a probability distribution over output classes. The output of this distribution is defined as in Eq (5)

$$\begin{array}{c} p\left(a==c|b\right)=\frac{e^{b_{k}}}{\sum_{j}e^{b_{j}}}, \end{array}$$ (5)

where b and c represents the probabilities that are retrieved from the softmax layer and one of the classes in dataset, respectively. Sample accuracy/loss plot with good-fit convergence achieved during training on dataset-1 (Ref. to section) is shown in Fig 3.

## Experimental setup

### Datasets

**Fig 4:**
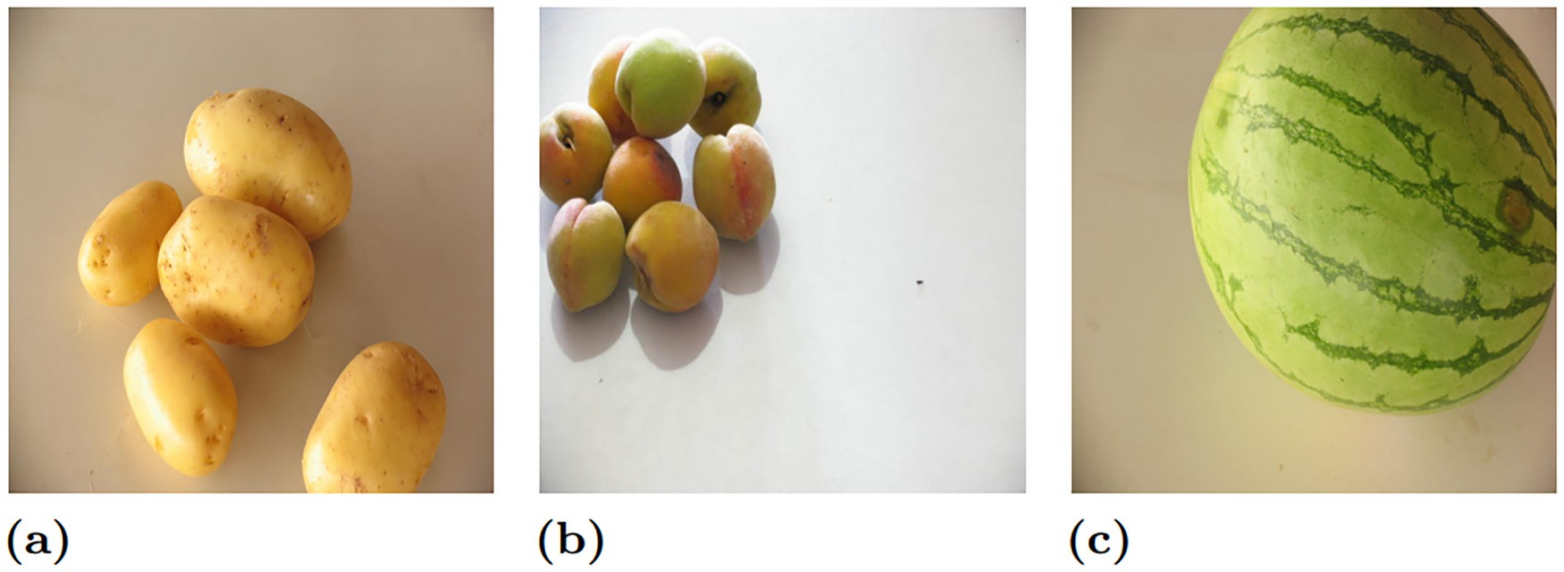
Sample fruits images abstracted from Dataset D1. Note that (a), (b), and (c) denote “potato”, “diamond peach” and “watermelon” fruits classes, respectively.

**Fig 5:**
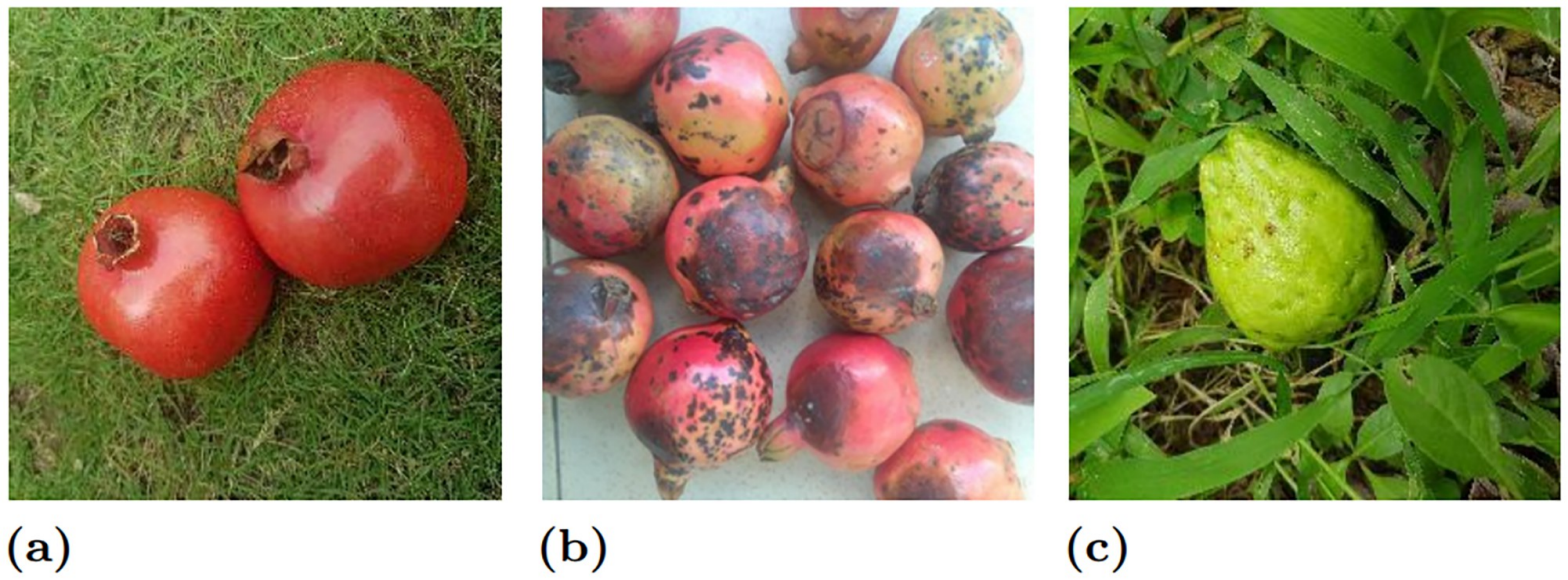
Sample fruits images abstracted from Dataset 2 (D2). Note that (a), (b), and (c) denote “pomegranate good”, “pomegranate bad” and “guava good” fruits classes, respectively. Republished from [51] under CC BY license.

**Fig 6:**
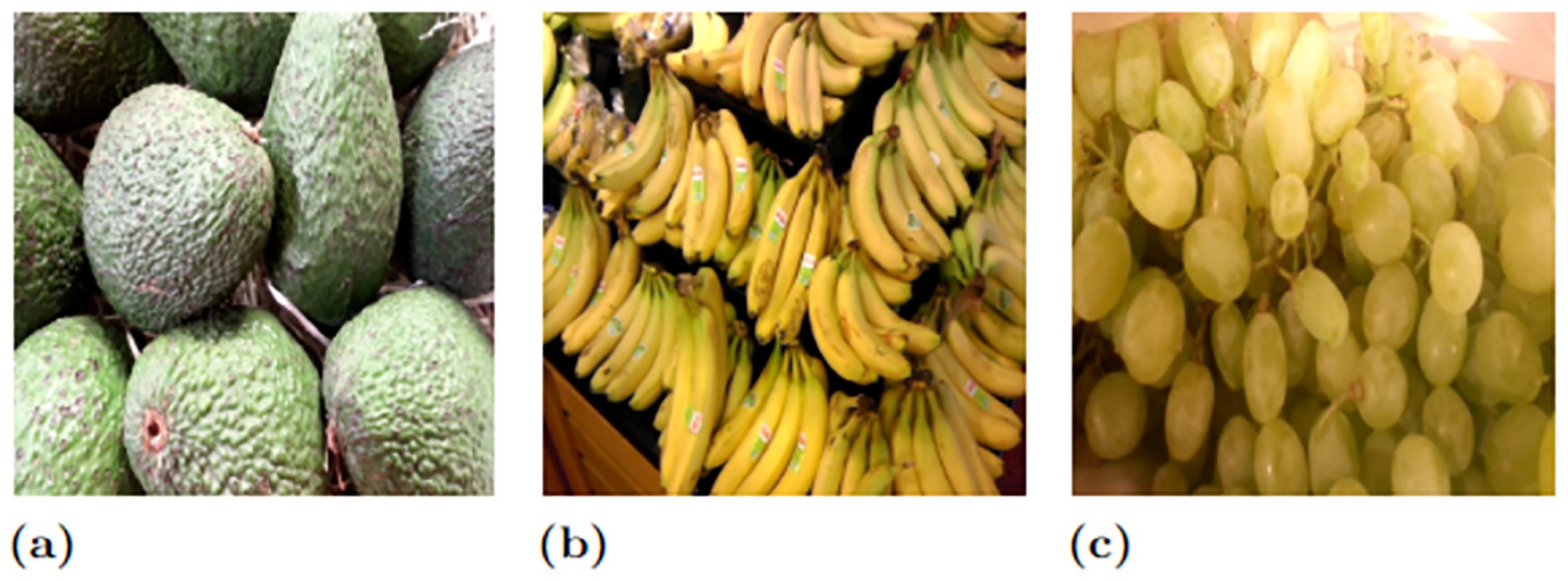
Sample fruits images abstracted from Dataset 3 (D3). Note that (a), (b), and (c) denote “Avacado”, “Banana” and “Grape” fruits classes, respectively. Note that the fruit image used here is Republished from [44] under CC BY license, with permission from [Georg Waltner], original copyright[2022].

We collect three different kinds of fruit-related datasets (Dataset 1, Dataset 2, and Dataset 3) to perform the fruit classification. Sample images are shown in Figs 4–6 for Dataset 1, Dataset 2, and Dataset 3, respectively.

**Dataset 1 (D1)** [50]: This is a publicly available fruit and vegetable dataset, which contains 15 classes. Each class contains at least 75 images, resulting in 2,633 images in total. These images were collected at a resolution of 1,024x768 pixels on different date and time. The dataset is freely available from [50].

**Dataset 2 (D2)** [51]: This is an Indian fruit dataset, which contains 12 classes. This is a balanced dataset, where each class has 1,000 images, resulting in 12, 000 images in total. Each image is taken with different angles, backgrounds and lighting conditions. The dataset is available publicly from [51].

**Dataset 3 (D3)** [44]: This is the largest fruit and vegetable dataset having classes at various levels: 53 classes at first level, 81 fine classes at second level and 125 classes at third level. In this work, we consider the 53 classes from the first level for fruit and vegetable classes corresponding to general food items. It consists of 15,737 images in total. This dataset is available from the website [44].

### Implementation

Our proposed method is implemented in Python [52] using Keras [53]. The hyper-parameters used in our work are presented in Table 2. We split each dataset to the train and test sets with a ratio 70:30 per category. Five different random train/test splits are used for each dataset to report the final averaged performance. To prevent model from over-fitting during training, we set 20% of train set for validation and change the learning rate value for each epoch as defined in Eq (6).

$$\begin{array}{c} \alpha_{n}=\alpha_{0}*0.4^{\frac{1+epoch(n)}{4}} \end{array}$$ (6)

where *α*_*n*_, *α*_0_ and epoch(n) represent the learning rate at *n*^*th*^ epoch, initial learning rate, and the current epoch number respectively.

Furthermore, to make comparison on the level ground, we also implemented four latest DL methods on the same computer with Tesla-P100 GPU with 16GB RAM.

**Table 2:** Detailed hyper-parameters used in our study.

Parameters	Value
Image size	224 × 224
Batch size	64
Epoch	60
Rotation range	90
Height shift range	0.2
Width shift range	0.2
Shear range	0.2
Vertical flip	True
Optimizer	Adam
Validation spit	0.2
Learning rate (*α*_0_)	0.0001
Loss	Categorical cross-entropy
Zoom range	0.2
Channel shift range	20
Horizontal flip	True

### Evaluation metrics

We use seven evaluation metrics (Eqs (7) to (13)) to evaluate the performance of our proposed model. These are calculated using confusion matrix from classifications. The confusion matrix tabulates the actual classes versus predicted classes. The diagonal of confusion matrix represents the correctly classified instances during classifications.

$$\begin{array}{c} P_{a}=\frac{TP_{a}}{TP_{a}+FP_{a}}, \end{array}$$ (7)

$$\begin{array}{c} R_{a}=\frac{TP_{a}}{TP_{a}+FN_{a}}, \end{array}$$ (8)

$$\begin{array}{c} F1_{a}=2\times \frac{P_{a}\times R_{a}}{P_{a}+R_{a}}, \end{array}$$ (9)

$$\begin{array}{c} ACC=\frac{TP_{a}+TN_{a}}{TP_{a}+TN_{a}+FP_{a}+FN_{a}} \end{array}$$ (10)

where *TP*_*a*_, *TN*_*a*_, *FP*_*a*_ and *FN*_*a*_ represent true positive, true negative, false positive and false negative for class ‘a’. Similarly, *P*_*a*_, *R*_*a*_ and *F*1_*a*_ represent precision, recall and F1-score for class ‘a’.

$$\begin{array}{c} Kappa-score=\frac{p_{o}-p_{e}}{1-p_{e}}, \end{array}$$ (11)

where *p*_*o*_ is the observed agreement ratio and *p*_*e*_ is the hypothetical probability of chance agreement.

$$\begin{array}{c} MAF1=\frac{1}{\left|L\right|}\sum_{a\in L}F1_{a} \end{array}$$ (12)

$$\begin{array}{c} WAF1=\frac{1}{\sum_{a\in L}Supp\left(a\right)}\sum F1_{a}\times Supp\left(a\right) \end{array}$$ (13)

where L represents the set of classes, *Supp*(*a*) denotes the number of samples in class ‘a’ or support set for class ‘a’. MAF1 and WAF1 represents macro-average f1-score and weighted-average f1-score respectively.

## Results and discussion

### Comparison with latest DL methods

To reduce the potential bias in our companions, the results used for making comparison here are all from our own implementation of both our proposed method and the four latest DL methods used in [3, 4, 16] and [14]. The statistics of all experimental results are presented in Table 3. We report the averaged results over five runs for each method on each dataset for the evaluation metrics. Each row in the table corresponds to the evaluation results under the corresponding dataset.

**Table 3:** Precision, Recall, Kappa-score, MAF1, WAF1, and Acc. on D1, D2, and D3 using averaged metrics over five runs (%).

Dataset	Method	Precision	Recall	Kappa-score	MAF1	WAF1	Acc.
D1	Light-CNN [3]	88.60	87.40	86.50	86.69	87.20	87.49
	Fruit-CNN [16]	88.60	88.00	87.17	87.30	88.00	88.10
	CNN + Augmentation [4]	92.20	91.40	90.77	90.94	91.42	91.43
	MobileNetV2+TL [14]	94.23	92.10	91.88	92.38	92.03	92.47
	**Ours**	**95.80**	**96.00**	**95.43**	**95.74**	**95.75**	**95.75**
D2	Light-CNN [3]	93.60	93.00	92.42	93.03	93.03	93.05
	Fruit-CNN [16]	93.40	93.00	92.40	93.04	93.04	93.03
	CNN + Augmentation [4]	94.60	94.00	93.43	93.78	93.78	93.96
	MobileNetV2+TL [14]	95.85	95.87	95.48	95.83	95.83	95.83
	**Ours**	**96.80**	**96.80**	**96.43**	**96.74**	**96.74**	**96.72**
D3	Light-CNN [3]	67.77	65.00	63.48	61.20	64.93	65.04
	Fruit-CNN [16]	74.33	74.01	73.18	69.84	73.91	74.08
	CNN+Augmentation [4]	88.50	88.20	87.71	87.46	88.17	88.10
	MobileNetV2+TL [14]	94.98	94.89	94.81	94.07	94.87	94.81
	**Ours**	**96.67**	**95.67**	**96.12**	**95.96**	**96.23**	**96.24**

Note that MAF1, WAF1 and Acc. denote macro averaged F1-score, weighted averaged F1 score, and classification accuracy respectively. Boldface represent the highest performance.

For D1, our method outperforms all four latest DL methods in terms of all performance indicators. Specifically, our method achieves the highest classification accuracy of 95.75% in comparison to the classification accuracies of 87.49% produced by the Light-CNN [3], 88.10% by the Fruit-CNN [16], 91.43% by the CNN+Augmentation [4] and 92.47 by the MobileNetV2+TL [14]. In this dataset, all existing methods achieve comparatively high performance, which might be due to the fact that images with homogeneous background make them easier to be classified. Among four existing methods, the MobileNetV2+TL [14] has the second-best performance, which is still inferior to ours by 3.28% in classification accuracy (95.75%) with a fewer trainable parameters (refer to Table 4. Similarly, our method outperforms the least-performing method (Light-CNN [3]) significantly with a margin of 8.26% in classification accuracy.

**Table 4:** Model parameters (’000) and running time (seconds) for each model.

Model	Total Params.	Trainable Params.	Training Time (s)	Inference time (ms)
Light-CNN [3]	1792	1792	1983.31	23.03
Fruit-CNN [16]	26277	26277	2611.01	24.61
CNN+Augmentation [4]	214	214	1799.95	13.24
MobileNetV2+Tl [14]	2627	369	1963.31	17.57
Ours	2424	166	1774.10	16.68

Note that the network parameters are rounded on thousands and training time and inference time are estimated on Tesla-P100 GPU with 16GB RAM with Dataset (D1).

For D2, our method outperforms all other four DL methods in terms of all six metrics. Since this dataset has a balanced number of samples in each fruit class (300 samples in each class in test set), all four DL methods achieve more than 93% accuracy and F1-scores (both WAF1 and MAF1 are equal as number of samples in each class are same) on this dataset. However, our method outperforms the second-best performing method (MobileNetV2+TL [14]) by 0.89%, and the least-performing method (Fruit-CNN [17]) by at least 3.69% better in classification accuracy with the least number of trainable parameters (refer to Table 4).

For D3, our method is the only outstanding performer, bettered all other DL methods by large margins in all evaluation metrics. This is likely due to the fact that the images in this dataset contain the heterogeneous background that require more useful features extracted from the image so as to properly separate the fruits from the backgrounds. The other DL methods only use the convolution features from the images for fruit classifications and hence are likely to be negatively affected by the heterogeneous backgrounds in the images. However, our method combines both the convolution and attention features extracted from images together, resulting in a higher performance against other DL methods. While observing results for dataset D3, we can speculate that the three CNNs (Light-CNN [3], Fruit-CNN [17], CNN-Augementation [4]) have the least performance in comparison to their own performance on other two datasets (D1 and D2). However, the MobileNetV2+TL [14] model has the consistent performance over all three datasets. The reason for this might be the features captured from the pre-trained model on ‘ImageNet’ dataset (which is the large image dataset with millions of images) while other three models are trained from scratch on the fruit-images only.

While looking at Table 4, our methods has the least trainable parameters compared to all four latest DL methods even though the total parameters in our model are more than the other two CNNs (Light-CNN and CNN+Augmentation). The reason for this is that we freeze all the layers in pre-trained MobileNetV2 and only train the few layers on the top of these layers (refer to Fig 1). With this strategy, we are able to reduce the training time (1774.10 seconds) compared to all other DL methods. This will makes our model easy for training and deploy in the new fruit image domains quickly. Also, the inference time for our model is second-best (16.68 milliseconds per image frame), which is good enough for applying our model in lightweight computing platform without compromising the classification accuracy.

Furthermore, we also present the accuracy of the four DL methods from respective article with their own experimental settings in Table 5 to further consolidate the results. Here, ‘Light-CNN’ model by Hossain et al. [3] used self-created fruit dataset with 5,946 images with ten classes, along with train/test split of ratio 85:15, batch size of 32, and epochs of 100. ‘Fruit-CNN’ by Mureşan et al. [16] used the publicly available dataset ‘Fruit-360’ and evaluated with various input settings such as grayscale image, RGB images, HSV images, batch size of 60, epochs of 50, fixed train/test split, and so on. ‘CNN+Augmentation’ by Joshep et al. [4] used the ‘Fruit-360’ dataset with the batch size of 128, epochs of 50, and ‘Adam’ optimizer along with extensive data-augmentation. MobileNetV2+TL used the self-created fruit dataset with 3,670 images. They used train/test split of 3,213 images in training and 457 images in testing with all image resized to 224 × 224. Because of the different experimental setup with different datasets in these existing studies, it is difficult to make a fair comparison between these methods and ours. However, on the level ground with the same evaluation matrices, our method should be a much improved higher performer in fruit classifications in different conditions.

**Table 5:** Reported accuracy of state of the art methods using classification accuracy (%).

Method	Acc.	dataset	Availability
Light-CNN [3]	85.43	Self-created [3]	Private
Fruit-CNN. [16]	95.23	Fruit-360 [16]	Public
CNN+Augmentation [4]	94.35	Fruit-360 [16]	Public
MobileNetV2+TL [14]	85.12	Self-created [14]	Private

Note that these accuracies are reported from the corresponding article, which are achieved based on the corresponding authors’ own experimental configuration and hyper-parameter settings.

### Comparison with pre-trained DL models

Here, we compare our proposed method with the existing deep learning models pre-trained on “ImageNet” dataset. We choose five pre-trained models (DenseNet-121 [37], NASNetMobile [38], VGG-16 [32], MobileNetV1 [34] and InceptionV3 [33]) for this comparison cohort as they are widely-used for fruit classification related applications. For instance, authors in [36] investigated the DenseNet [37] for oil palm fruit ripeness classification and produced the highest classification accuracy (86%). The transfer learning and fine-tuning of MobileNetV1 [34], InceptionV3 [33] and other CNNs were implemented in [14] for fruit classification. The VGG-16 [32] is still popular CNN model for the feature extraction and has been used in various domains ranging for medical image analysis [54] to fruit classification [39]. Among these five models, ‘NASNetMobile’ and ‘MobileNetV1’ are lightweight models while the rest are considered as the large and deep convolutional neural networks (CNNs). We believe that the comparative benchmarking of our model with both lightweight and heavy weight pre-trained models prove the robustness of our model. Our method outperforms all pre-trained models in comparison cohort in all performance metrics as shown in Table 6. For D1, the DenseNet-121 [37] is the second-best performing model with an accuracy of 94.53 while MobileNetV1 [34] has the least classification accuracy (86.69%) being lower by 9.06% with our method. The similar pattern is seen in other two datasets (D2 and D3), too, where the DenseNet-121 [37] is the second-best performing model in most of the performance metrics while our method is the best performer among all other contenders in most of the evaluation measures. It is interesting to note that our method being a lightweight method has comparable performance to those large and deeper CNNs (’VGG-16’, ‘InceptionV3’, ‘DenseNet-121’).

**Table 6:** Precision, Recall, Kappa-score, MAF1, WAF1, and Acc. on D1, D2, and D3 using averaged metrics over five runs (%).

Dataset	Method	Precision	Recall	Kappa-score	MAF1	WAF1	Acc.
D1	DenseNet121 [37]	94.50	94.50	94.11	93.88	94.47	94.53
	NASNetMobile [38]	88.50	87.01	86.21	86.03	87.32	86.69
	VGG-16 [32]	94.79	94.73	94.31	94.77	94.71	94.73
	MobileNetV1 [34]	88.14	86.7	85.65	85.57	85.75	86.69
	InceptionV3 [33]	90.17	89.71	88.29	89.24	89.72	89.71
	**Ours**	**95.80**	**96.00**	**95.43**	**95.74**	**95.75**	**95.75**
D2	DenseNet121 [37]	96.00	96.00	95.57	95.94	95.94	95.94
	NASNetMobile [38]	94.00	94.00	93.21	93.76	93.76	93.77
	VGG-16 [32]	96.22	96.11	95.75	96.10	96.10	96.11
	MobileNetV1 [34]	88.50	86.55	85.33	86.46	86.46	86.55
	InceptionV3 [33]	95.67	95.61	95.21	95.59	95.59	95.61
	**Ours**	**96.80**	**96.80**	**96.43**	**96.74**	**96.74**	**96.72**
D3	DenseNet121 [37]	95.00	94.00	93.89	92.30	94.00	94.10
	NASNetMobile [38]	86.50	85.00	84.61	81.93	85.75	85.05
	VGG-16 [32]	92.16	91.87	91.59	90.46	91.79	91.87
	MobileNetV1 [34]	94.46	94.64	94.22	94.35	94.33	94.41
	InceptionV3 [33]	91.47	90.79	90.46	89.59	90.45	90.79
	**Ours**	**96.67**	**95.67**	**96.12**	**95.96**	**96.23**	**96.24**

Note that MAF1 and WAF1, denote macro averaged F1-score, weighted averaged F1 score respectively. Boldface represent the highest performance.

### Ablative study of the proposed method

We present the efficacy of each individual features used in our work on D1. The experimental results are presented in Table 7. Both attention and convolution modules are responsible to improve the performance (Precision, Recall, MA_F1-score and Accuracy). The classification accuracy is 92.04% if using convolution module only, whereas it imparts 95.75% by including the attention module. Similar pattern can also be seen for other metrics (Precision, Recall, and MA_ F1). Hence, the surge of overall performance metrics is likely attributed to the synergic effect of the attention and convolution modules.

**Table 7:** Ablative study of our proposed model using average performance metrics (precision, recall, MAF1-score and accuracy) on D1.

Metrics	Attention (without)	Attention (with)
Accuracy	92.04	**95.75**
Precision	93.20	**95.80**
Recall	92.00	**96.00**
MAF1	91.80	**95.74**

Boldface indicates the highest performance.

### Class-wise discrimination of our proposed method

The statistics of our method on the class-wise discrimination on D1 is presented in Table 8 with the average of 5 runs. The class-wise Precision (Eq 7), Recall (Eq 8), and F1-score (Eq 9) are calculated for this purpose. It is clear that our method can produce high Precision (99.00%) and high Recall (100%) for fruit classes Cashew, Onion, and Watermelon. However, a low Precision and a high Recall are observed for fruit class Orange.

**Table 8:** Averaged class-wise Precision, Recall and F1-score of our model on test samples of D1 using over five runs (%).

Classes	Precision	Recall	F1-score
Agata potato	97.50	99.00	98.25
Asterix potato	**99.50**	99.00	99.25
Cashew	**99.00**	**100.00**	99.50
Diamond peach	97.75	81.00	88.50
Fuji apple	88.25	98.25	93.12
Granny smith apple	95.00	99.50	98.25
Honneydewmelon	**99.50**	99.00	99.25
Kiwi	98.00	96.00	97.00
Nectarine	89.75	90.75	90.25
Onion	**99.00**	**100.00**	99.50
Orange	81.75	**100.00**	89.50
Plum	98.25	97.00	97.50
Spanish pear	97.25	90.00	93.00
Taiti lime	98.25	89.00	93.25
Watermelon	**99.00**	**100.00**	**99.50**

Boldface indicates the highest performance.

### Qualitative analysis

**Fig 7:**
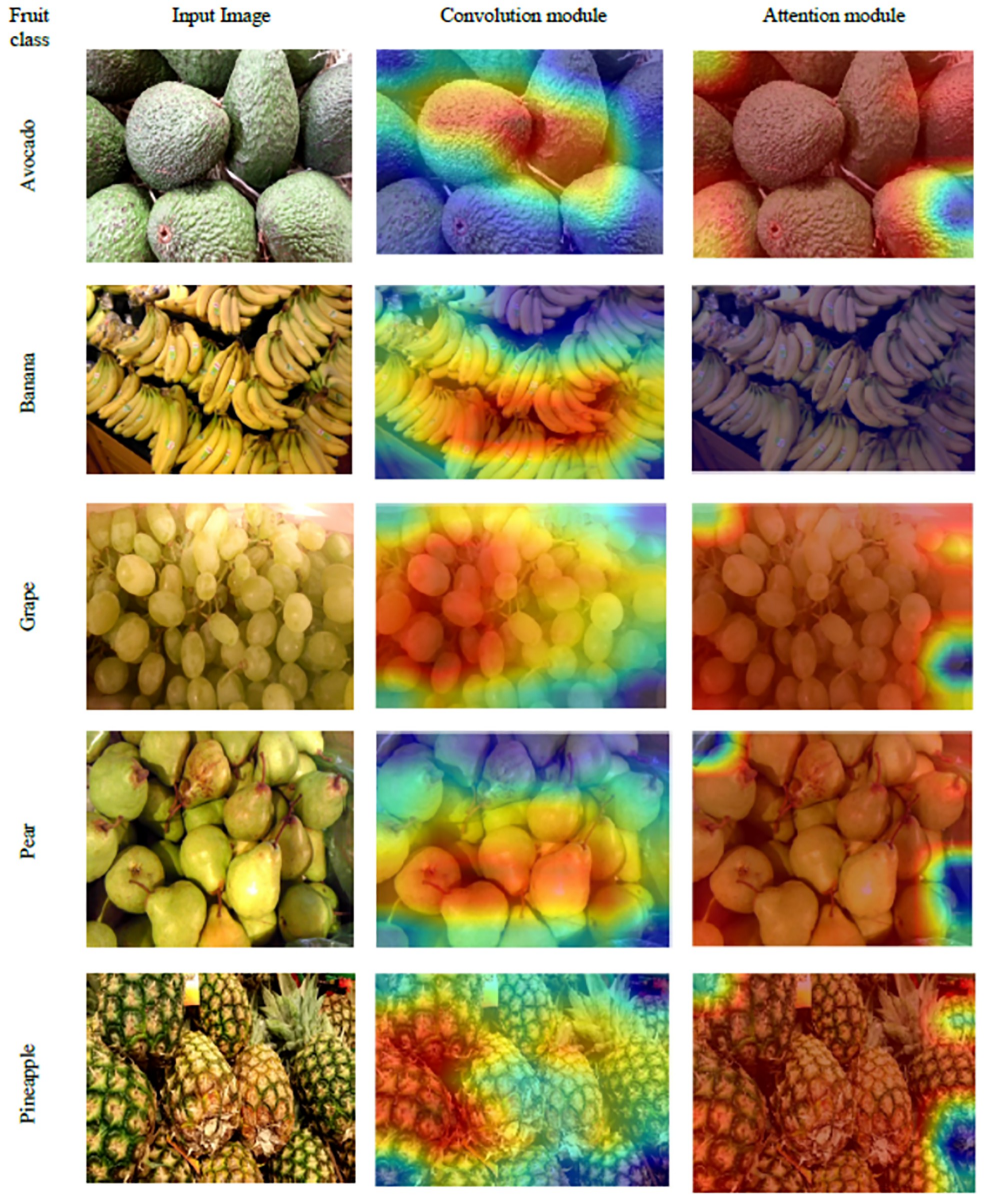
The GradCam [55] visualization examples for Fruits images from D3. Each row contains the original fruit image, its corresponding heatmaps extracted by Convolution module, and Attention module. Note that the fruit image used here is Republished from [44] under CC BY license, with permission from [Georg Waltner], original copyright[2022].

Here, we discuss the visual activation maps of selected images taken from dataset- 3 (D3) for the qualitative analysis of features. Given that our method consists of two main modules for feature extraction: convolution and attention, we list the sample original images along with a activation heatmap over the original image produced by convolution module and attention module in each row in Fig 7. While observing the first row, the feature map for Avocado fruits in convolution module is rendering at the centre of image while an attention module is more focused on specific salient regions such as corners and other regions than the convolution module. Similar patterns are observed in other fruits as well except the second row for banana fruit, where the attention module couldn’t capture any region of interest. This might be due to the uniform textures present in this image which don’t have any specific information to be captured by attention module. However, the convolution module is able to capture the sufficient features to distinguish the Banana fruits from other fruits as evident from higher classification accuracy of our model reported in Table 3. Thus, we believe that the convolution and attention modules impart the complementary information for better classification of fruits.

## Conclusion

In this paper, we presented a novel attention-convolution module based MobileNetV2 to classify the fruit images. Our method has achieved the stable classification accuracy of 95.75%, 96.74%, and 96.23% on Dataset 1 (D1), Dataset 2 (D2), and Dataset 3 (D3), respectively. Given the lightweight nature of our model, our method has a great potential to be adopted by industries closely related to the fruit growing and retailing or processing chain for automatic fruit identification and classifications in the future.

Our method has some limitations. First, our method relies on MobilenetV2 architecture. Hence, our model has not been tried with other user-defined lightweight backbone architectures. Second, our method uses online data augmentation only for our experiments. The performance of our model could be further improved by using or partly using other advanced offline data augmentation techniques, such as the Generative Adversarial Network (GAN). Third, the combination of features obtained from other layers of MobileNetV2 would be worth exploring for improving the performance of fruit classifications. We also need to explore how our method can efficiently operate in a mobile environment, or on a cutting-edge computing platform, particularly in an Internet of Things (IoT) environment.
